# Role of Zebrafish *Lbx2* in Embryonic Lateral Line Development

**DOI:** 10.1371/journal.pone.0029515

**Published:** 2011-12-22

**Authors:** Xiaowen Chen, Qiyong Lou, Jiangyan He, Zhan Yin

**Affiliations:** 1 Key Laboratory of Aquatic Biodiversity and Conservation of Chinese Academy of Sciences, Institute of Hydrobiology, Chinese Academy of Sciences, Wuhan, Hubei, China; 2 Graduate University of Chinese Academy of Sciences, Beijing, China; National University of Singapore, Singapore

## Abstract

**Background:**

The zebrafish *ladybird homeobox homologous gene 2* (*lbx2*) has been suggested to play a key role in the regulation of hypaxial myogenic precursor cell migration. Unlike their *lbx* counterparts in mammals, the function of teleost *lbx* genes beyond myogenesis during embryonic development remains unexplored.

**Principal Findings:**

Abrogation of *lbx2* function using a specific independent morpholino oligonucleotide (MO) or truncated *lbx2* mRNA with an engrailed domain deletion (*lbx2^eh-^*) resulted in defective formation of the zebrafish posterior lateral line (PLL). Migration of the PLL primordium was altered and accompanied by increased cell death in the primordium of *lbx2*-MO-injected embryos. A decreased number of muscle pioneer cells and impaired expression pattern of *sdf1a* in the horizontal myoseptum was observed in *lbx2* morphants.

**Significance:**

Injection of *lbx2* MO or *lbx2^eh-^* mRNA resulted in defective PPL formation and altered *sdf1a* expression, confirming an important function for *lbx2* in *sdf1a*-dependent migration. In addition, the disassociation of PPL nerve extension with PLL primordial migration in some *lbx2* morphants suggests that pathfinding of the PLL primordium and the lateral line nerve may be regulated independently.

## Introduction

The zebrafish lateral line system consists of a set of neuromasts, important mechanosensory organs which detect hydrodynamic variations and water currents, and their underlying neurons regularly arrayed along the surface of the head and body [1], [2], [3], [4], [5]. Neuromasts are composed of hair cells (HCs) and their characteristic surrounding cells, mainly mantle cells and supporting cells. HCs in the zebrafish lateral line system have a similar morphology and function to human HCs, and due to the external location, the zebrafish lateral line system has evolved into a strong model for investigation of HC toxicity, regeneration and protection, as well as screening for drugs to cure diseases associated with hearing loss in humans [6], [7], [8].

Development of the lateral line system in zebrafish has been studied at the embryonic stages, including control of the directional migration of the lateral line primordium, deposition and differentiation of neuromasts in the posterior lateral line (PLL) and mechanisms of HC polarity and regeneration. Until recently, several widespread signaling pathways including *sdf1a-cxcr4b/cxcr7*, *Fgf*, *Notch* and *Wnt* signaling have been suggested to synergistically function in the formation and maintenance of the lateral line system [9], [10], [11], [12], [13]. Many genes which are expressed in the migrating primordium and are putatively thought to be responsible for embryonic lateral line development are now being characterized [14]; however, genes which are expressed at undetectable levels in the PLL system, but which are able to affect the formation of PLL are largely elusive.

The zebrafish gene, *lbx2*, is one of several vertebrate counterparts of the ladybird family of homeobox genes in *Drosophila*, and has been proven to regulate myofibril formation and fin bud development [15]. *Lbx* gene family members are characterized by a N-terminal engrailed repressor domain. Amongst the vertebrates, mouse *lbx1* and *lbx2* were identified in 1999 [16] have been widely investigated. Murine *lbx1* has been reported to be necessary for myogenesis [17], [18], [19], [20], [21], neuronal development [22], [23], [24], [25], [26], [27], [28], [29] and neural crest-derived tissues [30]. Although apparent abnormalities are not detected in *lbx2*-null mice, *lbx2* may possibly be involved in ovarian development and folliculogenesis [31]. Further studies on the expression patterns and developmental roles of existing members of the *lbx* gene family are required to expand our knowledge of the evolution of these genes in vertebrates [32].

In this study, analysis of the phenotypes of *lbx2* morphants demonstrated that depletion of *lbx2* leads to PLL malformations in zebrafish. The similar PLL defects observed in both *lbx2* morphants and embryos injected with truncated *lbx2* mRNA with an engrailed domain deletion (*lbx2^ehI-^*) suggest a functional role for *lbx2* in development of the PLL. Integrity of the supporting cell and HC population in deposited neuromasts remained intact in *lbx2* morphants. However, an impaired expression pattern of *sdf1a* in the horizontal myoseptum was observed in *lbx2* morphants, as well as a defective migration pattern and increased cell death in the migrating PLL primordium. This study adds to the existing knowledge of the role of zebrafish *lbx2*, and deepens the understanding of lateral line development.

## Results

### Abnormal pattern of posterior lateral line neuromast deposition in *Lbx2* morphants

To study the function of *lbx2* in early zebrafish development, we significantly reduced lbx2 protein expression by employing a *lbx2* specific morpholino (MO), which targets the AUG start codon of *lbx2* mRNA to block translation. Firstly, we confirmed the specificity and efficiency of the *lbx2* MO. In order to test the efficiency of *lbx2* translational inhibition *in vivo*, we constructed an lbx2-EGFP reporter fusion protein construct, containing 60 bp of the 5′-UTR and the coding region for the first 66 amino acids of zebrafish lbx2 fused to the N-terminus of EGFP and a SV40 polyadenylation site, in the expression vector pEGFP-N1 (see Materials and Methods and Figure S1 for details). Transcription of *lbx2-EGFP* can be constitutively driven by the active human cytomegalovirus (CMV) promoter in embryos (Fig. S1A); therefore, we could detect translation of *lbx2-EGFP* by visualizing GFP fluorescence in embryos injected with linearized plasmid. When the *lbx2-EGFP* construct was co-injected with *lbx2* MO into zebrafish embryos, diminished GFP fluorescence was observed, indicating that translation of *lbx2-EGFP* can be effectively blocked by *lbx2* MO (Fig. S1B and S1C). Using an antibody against zebrafish *lbx2* which was developed in our laboratory, we detected lower lbx2 protein expression in *lbx2* morphants using Western blot analysis (Fig. S1D). Zebrafish *lbx2* has been suggested to play an essential role in myogenesis in the pectoral fin bud [15]. We observed an absence of *myoD*-positive cells in the pectoral fin bud region of most *lbx2* morphants, which could be rescued by co-injection of *lbx2* mRNA with the *lbx2* MO (Fig. S1E). In order to ensure that our experimental observations on the specific function of *lbx2* were accurate, we synthesized a mutated form of *lbx2* mRNA lacking the engrailed repressor domain, named *lbx2^eh-^* mRNA, for complementary analyses. The function of the truncated mRNA was indicated by depletion of *myod* expression in the pectoral fin bud of embryos injected with *lbx2^eh-^* mRNA (Fig. S1F–I). Taken together, *lbx2* MO and *lbx2^eh-^* mRNA provide specific and efficient tools for functional studies of *lbx2*.

We injected zebrafish embryos at the one-to-two cell stage with *lbx2*-MO and found that most *lbx2* morphants exhibited circling swimming behavior at the larval stage. Similar to *lbx2* morphants, *lbx2^eh-^* injected embryos also showed abnormal swimming behavior. Previous work has suggested that the mechanosensory lateral line system controls various types of swimming behavior in zebrafish [33]; therefore, we dissected the phenotype in order to analyze lateral line development in *lbx2* morphants.

**Figure 1:**
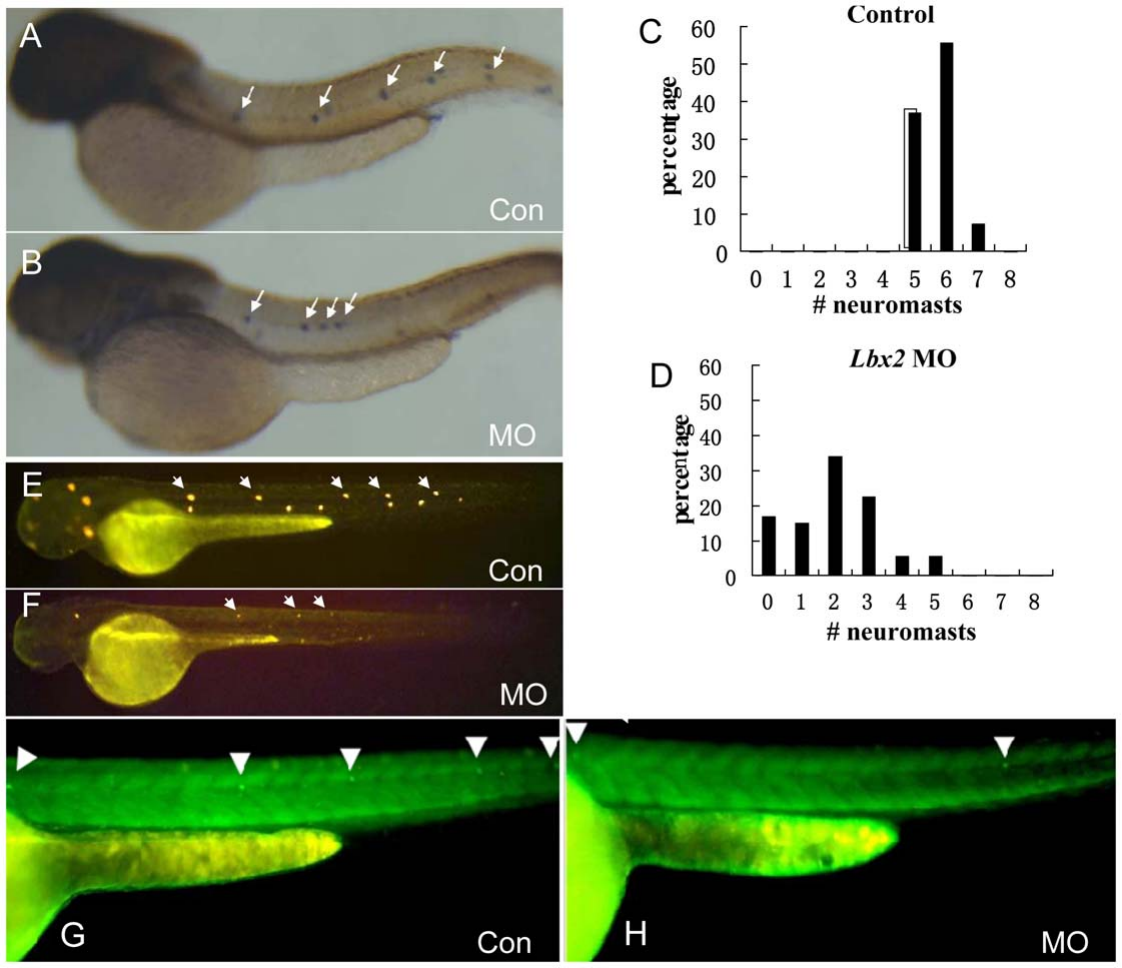
Abrogation of zebrafish *lbx2* results in malformation of the posterior lateral line (PLL). (A, B) Whole mount *in situ* hybridization using a *cldnb* antisense probe showing the normal phenotype in a control MO-injected embryo (A) and PLL neuromast abnormalities in a *lbx2* morphant (B). (C, D) Statistical distribution of the number of neuromasts per larva at 48 hpf counted unilaterally in controls (C) and *lbx2* morphants (D). (E, F) DiaAsp staining for functional, active deposited neuromasts in a control MO-injected embryo (E) and *lbx2* morphant (F). Please note the faint DiaAsp staining and reduced number of neuromasts (three stained neuromasts shown) in *lbx2* morphants, compared to the five clearly stained neuromasts in control larvae. (G, H) Compared to control embryos (G), injection of *lbx2^eh^* ^-^ mRNA lead to defective PLL formation in the SqET4 transgenic line (H). PLL hair cells are labeled with GFP; white arrows indicate clusters of hair cells in each lateral line neuromast.

Analysis of lateral line system development was conducted using the following experiments. Firstly, *Claudin b* (*cldnb*), a gene specifically expressed in PPL primordium and neuromast cells, was employed as a marker to observe the number and position of PLL neuromasts. The pattern of *cldnb* expression was examined in embryos at 48 hpf, at which stage neuromast sets have been deposited along the horizontal myoseptum to the tip of the tail during embryonic PLL primordium migration. As shown in Figure 1, 100% (57/57) of the embryos injected with control MO displayed the normal pattern of 5–7 *cldnb*-positive neuromasts; however, most *lbx2* MO injected embryos (39/41) showed decreased numbers of labeled neuromasts (average of three), and 15% (6/41) of the morphants lost all of the neuromasts (Fig. 1A–1D).

4-(4-diethylaminostyryl)-*N*-methylpyridinium iodide (DiAsp) can be incorporated by terminally differentiated HCs in lateral line neuromasts; therefore, DiAsp staining is an effective method for the detection of functional mature HCs [34]. At 48 hpf, neuromast HCs in control embryos were clearly stained with DiAsp, while faint DiAsp staining and decreased numbers of neuromasts were observed in *lbx2* morphants (Fig. 1E and 1F). Presence of the engrailed domain in lbx2 has been suggested to be critical for proper gene functioning [15]. In order to verify that the PLL defect observed in *lbx2*-deficient embryos was caused by functional depletion of *lbx2*, *lbx2^eh-^* mRNA was injected into SqET4 embryos. SqET4 is a recently developed transgenic zebrafish line expressing high levels of GFP in HC progenitors in the PLL [35]. An abnormal pattern of PLL neuromast deposition was observed in SqET4 embryos injected with *lbx2^eh-^* mRNA (Fig. 1G and 1H), similar to the PLL defects observed in *lbx2* morphants (Fig. 1B and 1F). These results suggest that impaired *lbx2* function specifically reduces the number of neuromasts and induces malformations in the PLL.

### The integrity of progenitor cells in deposited neuromasts and organization of the PLL primordium in *lbx2* morphants

**Figure 2:**
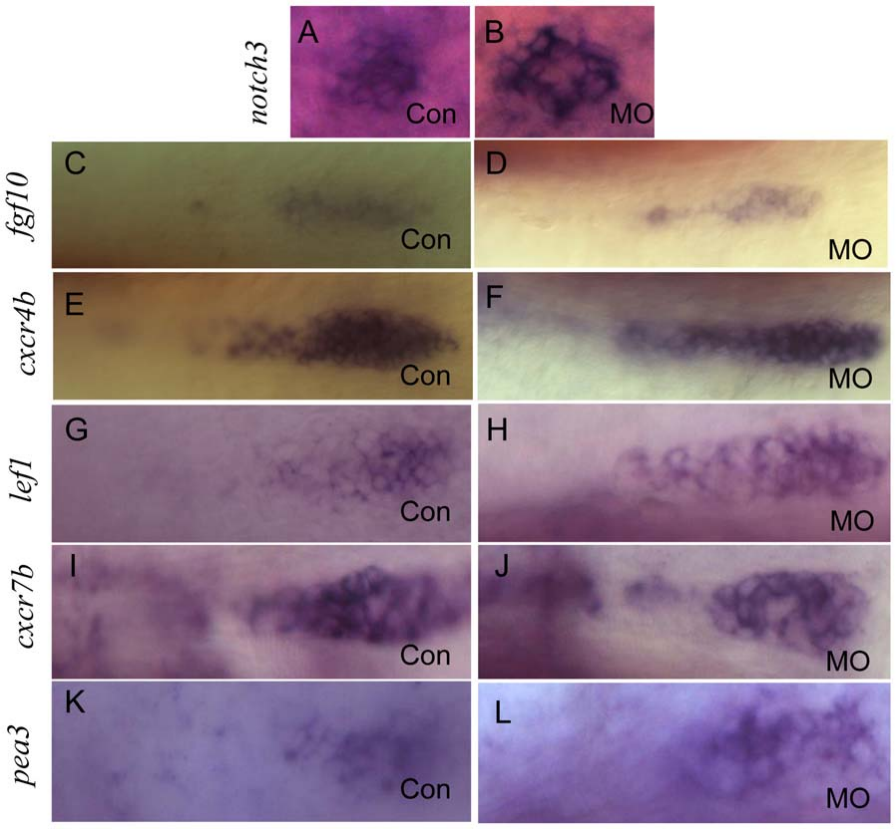
Integration of the migrating PLL primordia is not affected in *lbx2* morphants. (A, B) Whole mount *in-situ* hybridization for *notch3* indicating that compared to control embryos (A), the PLL hair cells of deposited neuromasts are not perturbed in *lbx2* morphants (B). (C, D) Compared control embryos (C), no difference in *fgf10* expression was evident in the protoneuromast rosettes of newly deposited neuromasts in *lbx2* morphants (D). (E, F) Compared to control embryos (E), there was no difference in *cxcr4b* expression in the protoneuromast rosettes of newly deposited neuromasts in *lbx2* morphants (F). (G, H) Compared to control embryos (G), there were no appreciable difference in *lef1* expression in the migrating PLL primordia in *lbx2* morphants (H). (I, J) Compared to control embryos (I), there were no appreciable difference in *cxcr7b* expression in the migrating PLL primordia in *lbx2* morphants (J). (K, L) Compared to control embryos (K), there were no appreciable difference in *cxcr7b* expression in the migrating PLL primordia in *lbx2* morphants (L). Embryos used in the assay were at 48 hpf stage.

It has been shown that *notch3* is expressed in support cells of PLL neuromasts [36]. Given the significant change in the neuromast number of *lbx2* morphants, we studied the support cell population in deposited neuromasts after *lbx2* depletion at 48 hpf. The number of *notch3*-expressing cells in the deposited neuromasts of *lbx2* morphants was comparable to control embryos (Fig. 2A and 2B). Using SqET10, a transgenic zebrafish expressing GFP in PLL supporting cells and nerves [35], and SqET4 embryos, we observed a similar pattern of GFP expression in the HCs and supporting cells of deposited neuromasts in *lbx2* morphants and control embryos (Fig. S2A and S2B). This data suggested that the supporting cells and HCs of deposited neuromasts were largely unaffected in *lbx2* morphants, indicating that *lbx2* is not essential for the survival and differentiation of supporting cells and HCs once neuromasts have been correctly deposited.

Initial formation of the PLL primordium requires cellular organization into protoneuromast rosettes. Migration of the PLL primordium begins when the leading two-to-three rosettes form; and then deposition onset occurs, followed by formation of a fourth rosette. Fgf ligands and *cxcr4b* are always expressed in the leading zone of the newly formed two or three rosettes, and control organization and polarity of the migrating PLL primordium [34]. Therefore, we performed whole-mount *in-situ* hybridization using antisense probes for *fgf10* and *cxcr4b*, and observed no obvious difference in the organization of newly deposited protoneuromast rosettes in *lbx2* morphants and controls (Fig. 2C–2F). These results reveal that *lbx2* is not involved in cellular organization or polarity of the PLL primordium. We also evaluated several other key components of pathways participating in PLL formation. *Lef1* are *pea3* are targets of wnt/beta-catenin and Fgf signaling, respectively; and *cxcr7* is a *sdf1a* receptor which is expressed in the posterior regions of the migrating PLL primordium. There was no appreciable difference in the expression of *lef1, pea3* and *cxcr7* in the migrating PLL primordia of *lbx2* morphants and control embryos (Fig. 2G–2L), indicating that although *lbx2* is essential for performance of lateral line system functions in response to dynamic external stimuli (Fig. 1 and 2), *lbx2* is not essential for regular signal integrity and cell differentiation in the PLL primordium.

### The lateral line nerve is not affected in *lbx2* morphants

**Figure 3:**
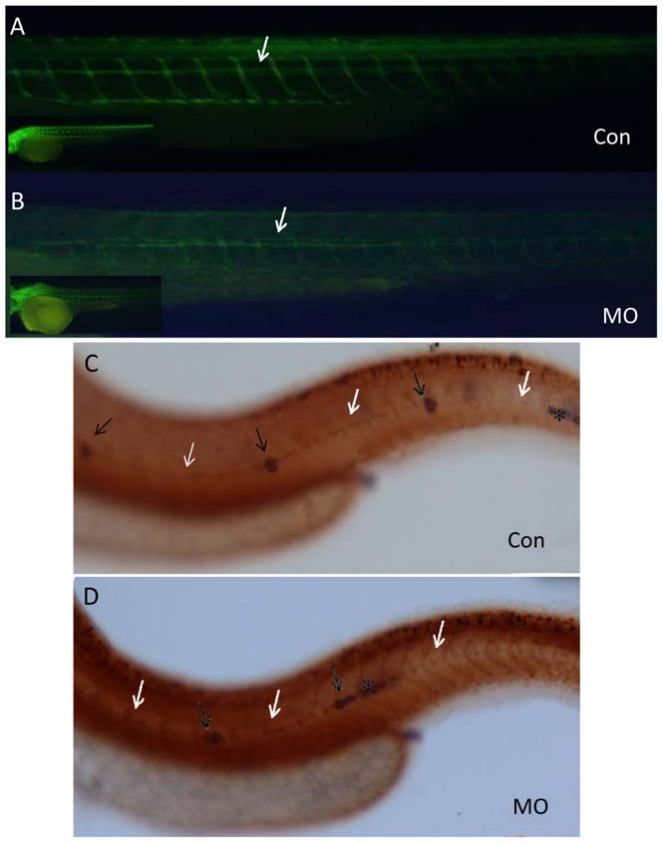
Disassociation of the PLL primodium and PLL nerve in *lbx2* morphants. (A, B) Labeling of the PLL nerve using an anti-acetylated α-tubulin antibody, indicating that the PLL nerve grew correctly in both control embryos (A) and *lbx2* morphants (B). (C, D) Immunohistochemical detection of the PLL nerve using an anti-acetylated alpha tubulin antibody to label PLL neuromasts and in situ hybridization using a *cldnb* antisense probe to label the primordia, revealing disassociation of the primodium and nerve in *lbx2* morphants (D), but not in control embryos (C). White arrows indicate the PLL nerve, black arrows show the deposited neuromasts and the black asterisk indicates the stalled primordium. Embryos used in the assay were at 48 hpf stage.

The PLL placode also generates PLL sensory neurons during primordium migration. Sensory growth cones remain associated with the migrating PLL primordium and form the PLL nerve. Recently, migration and development of the PLL nerve were shown to be stalled or misrouted during the malformation of PPL neuromasts when *sdf1a-cxcr4b* signaling is disrupted [36], [37]. In order to assess the possible function of *lbx2* in PLL nerve development, anti-acetylated-α-tubulin antibody was used to immunohistochemically label axons of the PLL or the lateral line nerve ganglion in *lbx2* morphants [36]. Unexpectedly, no obvious abnormalities were observed in the PLL nerves of most *lbx2* morphants at 48 hpf (Fig. 3A and 3B). By injecting the SqET10 line with *lbx2* MO, we found that the PLL axon could continue to extend in larva, even if neuromast deposition had stalled (Fig. 3C and 3D). The results suggest that PLL axons can be guided independently from PLL primordial migration via different molecular signaling systems.

### Impaired migration and increased cell death in the PLL primordium of *lbx2* morphants

**Figure 4:**
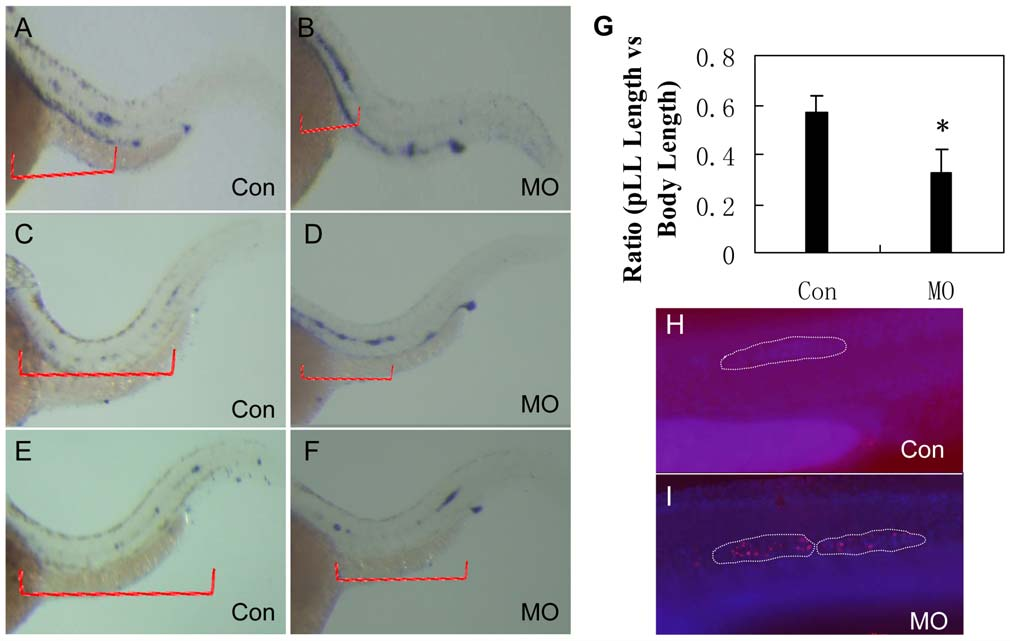
Migration of the PLL primordium is altered in *lbx2* morphants. Migrating PLL *atoh1a*-expressing hair cells in control embryos (A, C, E) and *lbx2* morphants (B, D, F) at 36 hpf (A, B), 40 hpf (C, D) and 48 hpf (E, F). (G) Analysis of the distance of PLL primordium migration in 48 hpf *lbx2* morphants and controls. (H, I) TUNEL assay coupled with DAPI staining, indicating elevated cell death in the slowly migrating PLL primordium and deposited neuromasts of *lbx2* morphants (I) compared with a similar region of the migrating PLL primordium in control embryos (H) at 36 hpf. Violet dots mark the migrating PLL primordium labeled with DAPI, stippled white lines indicate the zone of the PLL primordium and red dots indicate TUNEL positive cells.

We monitored the process of PLL primordium migration at several developmental stages from 30 to 48 hpf in control and *lbx2*-injected embryos by whole mount *in situ* hybridization using a probe for *atoh1a*, a marker of HCs in the migrating PLL primordium [38]. The leading edge of primI can be determined by *atoh1a* expressing cells. Ten embryos from the *lbx2*-MO injected group and control-MO injected group were examined at 36, 40 and 48 hpf. A significant reduction in the speed of primordium migration was observed in nearly all of the *lbx2* morphants tested (Fig. 4A–4G). In the most defective morphants, neuromasts were deposited in the anterior part of the migratory route (data not shown). In most of the moderately affected morphants, neuromasts were deposited in inappropriate locations as the primordium extended posteriorly at a reduced speed. Notably, similarly to other PLL primordium migration defects observed in a variety of zebrafish mutants and morphants [37], nearly all of the *lbx2* morphants possessed a correctly deposited first neuromast. To assess whether primordium migration defects could lead to increased cell death, we analyzed cell death in both control-MO and *lbx2*-MO-injected embryos at 36 hpf using whole-mount TUNEL staining. As shown in Figure 4H and 4I, the *lbx2* morphant primordium and deposited neuromasts (visualized as light violet dot clusters by DAPI staining) contained significantly higher numbers of TUNEL-positive cells (labeled as red dots) compared to control embryos. This suggests that stalled PLL primordium and/or misdeposited neuromasts in *lbx2* morphants may undergo apoptosis. Thus, overall disorganization of the PLL neuromasts in *lbx2* morphants could primarily be due to impaired primordium migration caused by loss of *lbx2* function.

### *Lbx2* deficiency in zebrafish affects expression of *sdf1a*

**Figure 5:**
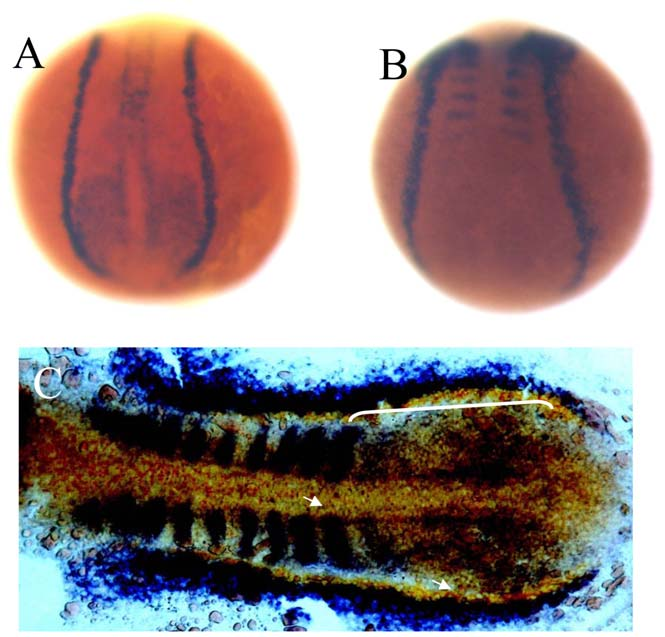
Overlapping expression domains of zebrafish *lbx2* and *sdf1a* during early development. (A, B) Expression patterns of *lbx2* (A) and *sdf1a* (B) at the tail bud stage (dorsal toward the top view). (C) *lbx2* (blue) and *sdf1a* (red) have an overlapping expression zone in the posterior lateral mesoderm and adaxial cells at the 10-somite stage (dorsal view). White bracket and arrows in C mark the overlapping zone of gene expression.

**Figure 6:**
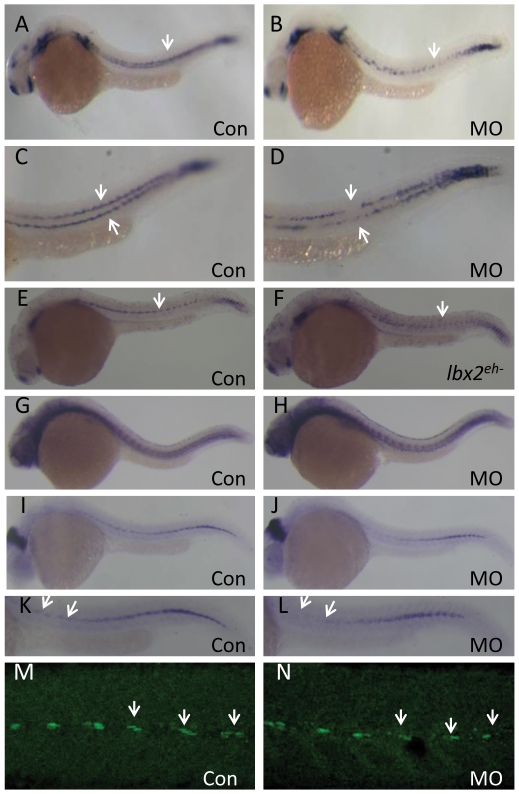
Abrogation of zebrafish *lbx2* results in a defective s*df1a* expression pattern in the horizontal myoseptum. (A, B) Compared with control embryos (A), a weak and discontinuous *sdf1a* expression pattern is observed in the horizontal myoseptum of *lbx2* morphants at 24 hpf (B; lateral view). (C, D) Magnified views of the expression patterns shown in A (C) and B (D). (E, F) Compared with *gfp* mRNA-injected control embryos (E), injection of *lbx2^eh^* ^-^ mRNA resulted in a defective *sdf*1a expression at 24 hpf (F). (G, H) Similar expression pattern of *tenascin C*, a marker of the horizontal myoseptum, in control embryos (G) and *lbx2* morphants (H) at 30 hpf. (I, J) Analysis of *eng2a* positive muscle pioneer cells in control embryos (I) and *lbx2* morphants (J) at 30 hpf. (K, L) Magnified views of the expression patterns shown in I (K) and J (L), showing decreased numbers of *eng2a* positive muscle pioneer cells in *lbx2* morphants (L) compared to control embryos (K). (M, N) Compared with control embryos (M), the numbers of 4D9 positive muscle pioneer cells was slightly reduced in *lbx2* morphants (N) at 30 hpf. White arrows indicate the hybridization or immunostaining signals. All images are lateral views.

Sdf1a is the main chemokine which drives correct migration of the PLL primordium via binding to cxcr4b. By 16 hpf, sdf1a is expressed in the posterior lateral mesoderm and adaxial cells. Sdf1a is synthesized by muscle pioneer cells and secreted into the horizontal myoseptum, and binds to cxcr4b in PLL primordium cells, thus guiding PLL migration [36]. Overlapping expression domains of zebrafish *lbx2* and *sdf1a* are present in the posterior lateral mesoderm at the tail bud stage (Fig. 5A and 5B) and in adaxial cells at the 10 somite stage (Fig. 5C). We have also observed partial co-localization of *lbx2* and *sdf1a* expression in adaxial cells and cells of the horizontal myoseptum at later developmental stages, as previously described [15], [36]. The existence of overlapping expression domains prompted a hypothesis of crosstalk between *lbx2* and *sdf1a*. We examined the expression pattern of *sdf1a* in *lbx2*-injected embryos at 24 hpf. In contrast to control embryos, most *lbx2* morphants had a weak and discontinuous pattern of *sdf1a* expression just before the PLL began to migrate (Fig. 6A–D). This defective *sdf1a* expression pattern could be replicated by injection of *lbx2^eh-^* mRNA into zebrafish embryos (Fig. 6E and 6F), and was reminiscent of the fragmentation which occurs when the PLL primordium migrates to the tip of the tail.

Impaired *sdf1a* expression along the horizontal myoseptum could be the result of damage to the anatomical integrity of the horizontal myoseptum [36], or loss of *sdf1a*-expressing pioneer cells. Using an antisense probe to *tenascin C* (a marker of the horizontal myoseptum [39], [40]), we measured the anatomical integrity of the zebrafish horizontal myoseptum. As shown in Figure 6G and 6H, the myoseptum was not disturbed in *lbx2* morphants. However, after a careful examination of muscle pioneer cells, using riboprobe hybridization of the muscle pioneer cell marker *eng2a* or 4D9 antibody staining [41], we found that fewer muscle pioneer cells formed in *lbx2* morphants (Fig. 6I–6N). These results suggest that the abnormal pattern of *sdf1a* expression in *lbx2* morphants may possibly be due to interference with the differentiation of *sdf1a*-expressing cells in the horizontal myoseptum after abrogation of *lbx2*. Although expression of *lbx2* was never observed in the PLL primordium, our results suggest that *lbx2* may interfere with the stereotypical pathway of the migrating PLL primordium via dysregulated secretion of sdf1a along the myoseptum.

## Discussion

*Ladybird homeobox (Lbx)* genes are a group of homeodomain-containing genes related to the *Drosophila* ladybird genes [42]. During post-gastrulation limb and fin development in zebrafish, *lbx1* and *lbx2* display a conserved expression pattern in a subset of hypaxial myoblasts. Before hypaxial myoblasts migrate to the limb/fin buds, they transition from epithelial to mesenchymal cells in the dermomyotome. Loss of *lbx1* and *lbx2* in animal models results in a lack of limb/fin musculature attributed to muscle progenitor cells migration defects [15], [18], [43], [44], [45]. Knockout studies in mice suggest that *lbx1* is involved in interpreting the signals which guide appendicular muscle precursor migration [18], [43], [44], and *lbx2* is involved in development of the pectoral fin bud muscle in zebrafish [15]. The expression pattern of *lbx2* has been characterized in zebrafish; however, the functions of *lbx2* in afferent neural tissue development during early embryogenesis remain largely unknown.

Previous studies have suggested that a number of cellular or molecular relationships between muscle development and lateral line development exist [41], [46], [47]. These studies have mainly focused on genes (such as *met* and *prox1*), whose transcripts are present in both muscle tissue and the lateral line system. However, this study reveals a novel function for *lbx2* in the control of PLL migration via the modulation of *sdf1a* expression. The co-expression of *lbx2* and *sdf1a* in the early embryonic stages has already been observed. This study demonstrates that *lbx2* is involved in maintenance of the precise expression pattern of *sdf1a* in the horizontal myoseptum. Thus, the loss of *lbx2* function, due to injection of *lbx2* MO or *lbx2^eh-^* mRNA, lead to abnormalities in the expression pattern of *sdf1a* and affected the ability of *sdf1a* to guide migration of the PLL primordium. As *lbx2* contains an engrailed domain, it is normally considered to be a transcriptional repressor. Our results suggest that the putative repressor domain of zebrafish *lbx2* is required to maintain the correct pattern of *sdf1a* expression in the horizontal myoseptum. Therefore, *sdf1a* is unlikely to be a direct repression target of *lbx2*. Previous studies have suggested zebrafish *lbx2* plays a role in muscle precursor differentiation and myofibril formation [15], and we observed decreased numbers of *eng2a* or 4D9 positive muscle pioneer cells in *lbx2* morphants (Fig. 6), demonstrating that zebrafish *lbx2* plays an important role in the regulation of *sdf1a*-expressing muscle pioneer cell differentiation. This study strongly suggests that regulation of lateral line primodium migration by the *sdf1a-cxcr4b*-signaling cascade requires expression of *lbx2*, even though expression of *lbx2* cannot be detected in the lateral line.

In parallel with our observation that depletion of *lbx2* leads to defective migration of the PLL primodium (Fig. 1), the neuromasts deposited along the migration path remained largely intact (Fig. 2 and Fig. S2). The overall malformation of the PLL in *lbx2* morphants could result from elevated cell death in misplaced or stalled neuromasts at aberrant locations along the migration route (Fig. 4). It has been suggested that both the PLL primordium and axons are guided by *sdf1a-cxcr4b* signaling [36], [37] or signals present in the migrating PLL primordia [48]. We extended our studies to the PLL nerve in *lbx2* morphants. Interestingly, the growth and extension of the PLL nerve in *lbx2* morphants appeared normal, even in the presence of a high level of misdeposition or stalling of the primordia (Fig. 3C–3D). This indicates that extension of PLL axons is guided independently of PLL primordium migration, suggesting that formation of the PLL nerve may not ultimately depend on the *sdf1a-cxcr4b* system or movement of the PLL primordium. Although we still cannot define the exact cause of the abnormal swimming behavior observed in *lbx2* morphants, it is possible that sensory organ defects or malformation of the pectoral fin, rather than defects in the PLL, may lead to abnormal swimming behavior.

In conclusion, this study describes a previously unrecognized role for the involvement of *lbx2* in zebrafish PLL formation, via a regulatory function in muscle development. Comparative analysis and molecular dissection of the migrating PLL could provide us with more information about the early history of animal evolution, as three *lbx* genes, *lbx1a*, *lbx1b* and *lbx2*, are present in the genome of teleost fish such as zebrafish [15], [32].

## Materials and Methods

### Zebrafish maintenance

Wild-type zebrafish, and the SqET4 and SqET10 trap lines specifically expressing EGFP in the lateral line (gifts of Prof. V. Korzh at the Institute of Molecular and Cell Biology, Singapore), were maintained as previously described [49]. Embryos were collected by natural matings of zebrafish adults and incubated in egg water at 28.5°C [49]. Embryos at different developmental stages were staged by hours post fertilization (hpf) [50].

### Whole mount *in situ* hybridization and antibody staining

For whole mount *in situ* hybridization, embryos older than 48 hpf were incubated in egg water containing 0.003% 1-phenyl-2-thiourea (PTU, Sigma, St. Louis, MO, USA) from 12 hpf onwards to prevent pigmentation. Embryos reaching the desired developmental stages were fixed in 4% PFA/PBS overnight at 4°C, dehydrated in 100% methanol and stored at −20°C before use. Whole-mount *in-situ* hybridization procedures (WISH) and double *in-situ* hybridization were performed as previously described [51]. Antisense probes for *cldnb, fgf10, sdf1a, cxcr4b, cxcr7, tenascin C, atoh1a, lbx2, lef1, eng2a, pea3* and *notch3* were synthesized using T7 or SP6 RNA polymerases and labeled with digoxigenin-UTP or fluorescein-UTP (Roche, Mannheim, Germany). The detailed whole-mount immunohistochemistry staining procedure has previously been described [52]. The antibody against acetylated-α-tubulin (Santa Cruz Biotechnology, CA, USA) and 4D9 (Developmental Studies Hybridoma Bank, Iowa City, IA, USA) were used at 1∶200 or 1∶100 dilution. The primary rabbit polyclonal antibody against zebrafish lbx2 used for Western blotting was developed in our laboratory, purified and used at 1∶50 dilution. For double staining using the *cldnb* antisense probe and anti-acetylated α-tubulin antibody, *in situ* hybridization was followed by immunohistochemical staining.

### *In-vitro* mRNA synthesis

Wild-type *lbx2* cDNA or truncated *lbx2* cDNA lacking the repressor engrailed domain [15] were cloned into the pSP64-T vector (Promega, Madison, WI, USA). Capped *lbx2* and *lbx2^eh-^* mRNAs were transcribed from the linearized plasmids using the mMACHINE *in-vitro* transcription kit (SP6; Ambion, Austin, TX, USA) according to the manufacturer's instructions.

### Embryo micro-injection

The sequence of the *lbx2* translation-blocking morpholino oligonucleotide (MO) was 5′-ctactggaggtcgagatttctgtac-3′ (ATG complementary sequence underlined). *lbx2*-MO (0.5 mM), standard-MO (0.5 mM) or 50 ng/ul *lbx2* wild-type or truncated mRNA were dissolved in sterile double-distilled water containing phenol red and injected into one-two-cell stage zebrafish embryos using a Harvard micro-injector (Harvard Apparatus, Holliston, MA, USA). The effectiveness of zebrafish *lbx2* MO translational inhibition was tested by detection of lbx2-EGFP green fluorescent fusion protein *in vivo*, which is described in more detail in Supplementary Figure S1.

### DiAsp staining

DiAsp (4-Di-2-Asp, Sigma D3418) was dissolved in double distilled water at 500 mM and stored at 4°C. To label the neuromast hair cells, live 48 hpf morphants and control embryos were incubated in egg water containing 5 mM DiAsp for exactly 5 min, rinsed several times with fresh egg water for 5 min as previously described [53], and visualized using fluorescence microscopy.

### TUNEL staining and measurement of PLL length

The TUNEL (Terminal dUTP Nick-End-Labeling) assay to detect cell death in zebrafish embryos was carried out as previously described [54], then after several washes in PBST, the embryos were transferred to DAPI solution to visualize the nuclei of the PLL primordium.

Ten randomly selected embryos (over 48 hpf) from morphants and controls were analyzed. The distance between the first neuromast and the end of primordium relative to the length of the body axis curvature was measured using ImageJ software (NIH). Significance analysis was conducted via Student's t-test in Excel.

## Supporting Information

Figure S1 **Efficiency of the** ***lbx2*** **morpholino.** (A) A test *lbx2-EGFP* construct was created containing 60 bp of the 5′ UTR and the first 66 amino acid coding sequence of *lbx2* cDNA fused to the N-terminus of EGFP, driven by the CMV promoter. The sequence of *lbx2* MO is complementary to the 1–24 bp region of zebrafish *lbx2* cDNA. (B) Live embryos at the 50% epiboly stage. Embryos co-injected with 25 ng *lbx2-EGFP* DNA and 5 ng control MO expressed green fluorescent fusion protein (left), which was inhibited by co-injection of 2 ng *lbx2* MO (right). (C) Translation of *lbx2-EGFP* in live embryos was inhibited by co-injection of *lbx2* MOs. (D) The *lbx2* protein level in *lbx2*-MO-injected embryos was drastically lower than control MO-injected embryos at 30 hpf (E). Absence of *MyoD* expression in the pectoral fin bud of *lbx2* morphants at 48 hpf, which could be rescued by co-injection of *lbx2* mRNA. Arrowhead indicates *MyoD* expression in the pectoral fin bud area. (F–I) Injection of *lbx2^eh^* ^-^ mRNA dramatically inhibited *MyoD* expression in pectoral fin muscle precursors at 30 hpf (G) and 36 hpf (I), compared to *gfp* mRNA-injected control embryos (F, H).
(TIF)

Figure S2 **PLL cells in the newly deposited neuromasts of** ***lbx2*** **morphants appear normal. (A–B)** Hair cells of PLL neuromasts labeled with GFP in the SqET4 transgenic zebrafish line. The pattern and numbers of PLL hair cells in newly deposited neuromasts was similar in control embryos (A) and *lbx2* morphants (B) at 48 hpf. (C, D) Fluorescence images of SqET10 embryos indicating that the supporting cells and lateral line nerve in newly deposited neuromasts of embryos injected with control MO (C) or *lbx2* MO (D) are similar at 48 hpf. The white arrowhead indicates HCs in deposited neuromasts.
(TIF)
