# Correction: Role of Zebrafish *Lbx2* in Embryonic Lateral Line Development

**DOI:** 10.1371/annotation/a245f079-8184-47da-93d5-7d9e34e54279

**Published:** 2012-05-23

**Authors:** Xiaowen Chen, Qiyong Lou, Jiangyan He, Zhan Yin

There was an error in the text in the "Embryo micro-injection" section of Materials and Methods. The correct lbx2-MO sequence is the reversed version of the published sequence in the text. It is 5'-CATGTCTTTAGAGCTGGAGGTCATC-3'. 

