# Supplementary figures and images for: Role of Zebrafish Lbx2 in Embryonic Lateral Line Development
Source: PLoS One. 2011 Dec 22;6(12):e29515. doi: 10.1371/journal.pone.0029515 (PMC3245281; doi:10.1371/journal.pone.0029515)

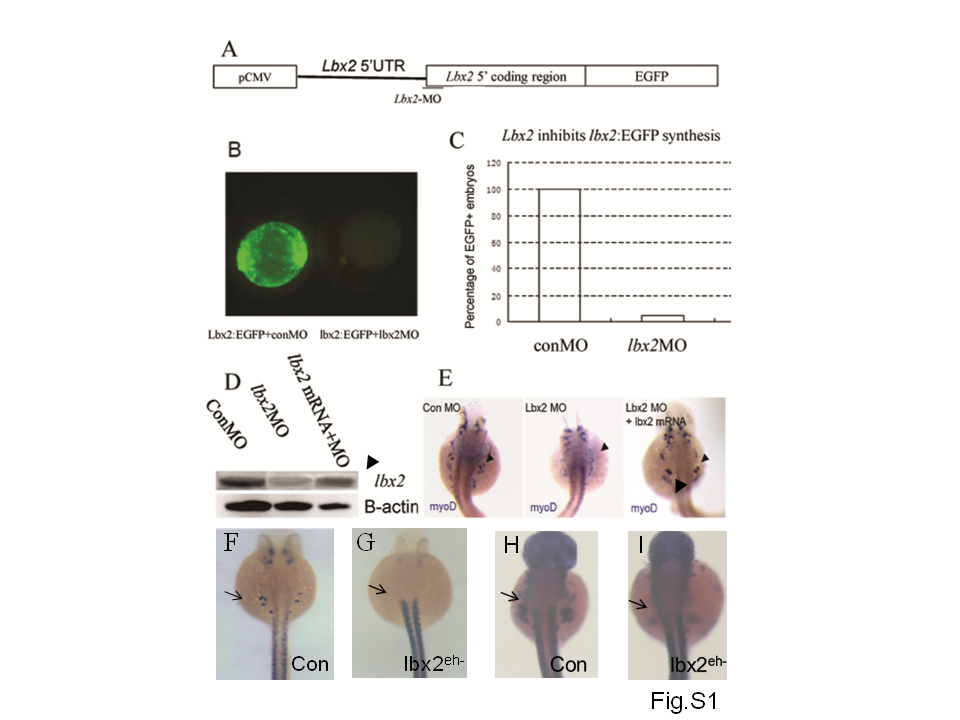

Supplement: Figure S1 — Efficiency of the lbx2 morpholino. (A) A test lbx2-EGFP construct was created containing 60 bp of the 5′ UTR and the first 66 amino acid coding sequence of lbx2 cDNA fused to the N-terminus of EGFP, driven by the CMV promoter. The sequence of lbx2 MO is complementary to the 1–24 bp region of zebrafish lbx2 cDNA. (B) Live embryos at the 50% epiboly stage. Embryos co-injected with 25 ng lbx2-EGFP DNA and 5 ng control MO expressed green fluorescent fusion protein (left), which was inhibited by co-injection of 2 ng lbx2 MO (right). (C) Translation of lbx2-EGFP in live embryos was inhibited by co-injection of lbx2 MOs. (D) The lbx2 protein level in lbx2-MO-injected embryos was drastically lower than control MO-injected embryos at 30 hpf (E). Absence of MyoD expression in the pectoral fin bud of lbx2 morphants at 48 hpf, which could be rescued by co-injection of lbx2 mRNA. Arrowhead indicates MyoD expression in the pectoral fin bud area. (F–I) Injection of lbx2eh - mRNA dramatically inhibited MyoD expression in pectoral fin muscle precursors at 30 hpf (G) and 36 hpf (I), compared to gfp mRNA-injected control embryos (F, H). (TIF) [file pone.0029515.s001.tif]

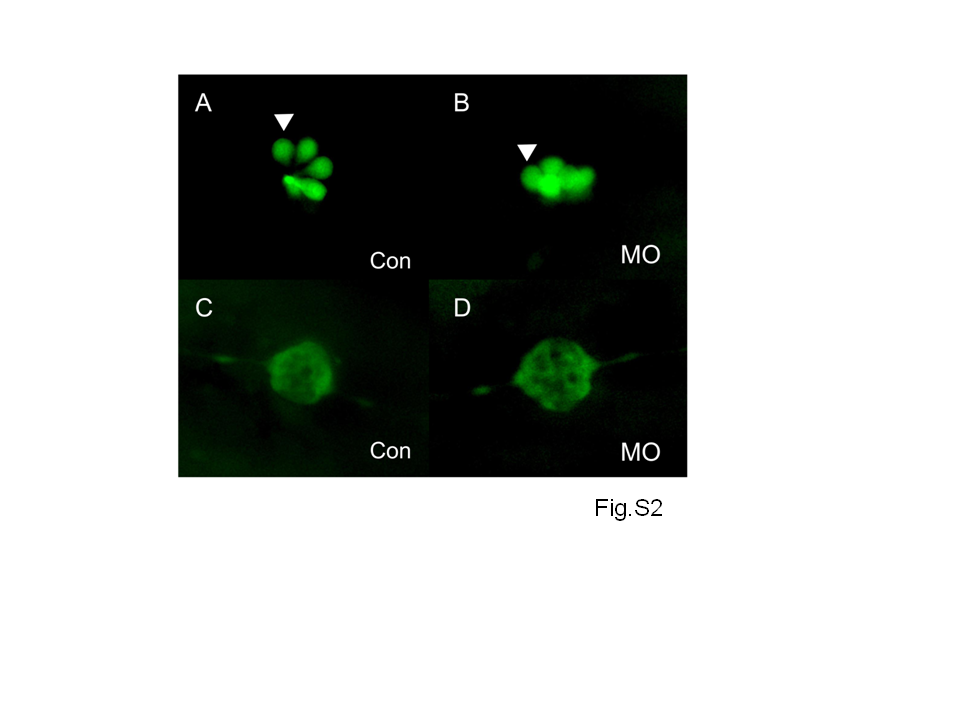

Supplement: Figure S2 — PLL cells in the newly deposited neuromasts of lbx2 morphants appear normal. (A–B) Hair cells of PLL neuromasts labeled with GFP in the SqET4 transgenic zebrafish line. The pattern and numbers of PLL hair cells in newly deposited neuromasts was similar in control embryos (A) and lbx2 morphants (B) at 48 hpf. (C, D) Fluorescence images of SqET10 embryos indicating that the supporting cells and lateral line nerve in newly deposited neuromasts of embryos injected with control MO (C) or lbx2 MO (D) are similar at 48 hpf. The white arrowhead indicates HCs in deposited neuromasts. (TIF) [file pone.0029515.s002.tif]
